# Intermittency, Moments, and Friction Coefficient during the Subcritical Transition of Channel Flow

**DOI:** 10.3390/e22121399

**Published:** 2020-12-11

**Authors:** Jinsheng Liu, Yue Xiao, Mogeng Li, Jianjun Tao, Shengjin Xu

**Affiliations:** 1 Applied Mechanics Laboratory, School of Aerospace Engineering, Tsinghua University, Beijing 100084, China; ljs16@mails.tsinghua.edu.cn; 2 Center for Applied Physics and Technology, Department of Mechanics and Engineering Science, College of Engineering, Peking University, Beijing 100871, China; xiaoyue904@pku.edu.cn; 3 Department of Mechanical Engineering, University of Melbourne, Victoria 3010, Australia; mogengl@student.unimelb.edu.au

**Keywords:** subcritical transition, channel flow, turbulence fraction, moment

## Abstract

The intermittent distribution of localized turbulent structures is a key feature of the subcritical transitions in channel flows, which are studied in this paper with a wind channel and theoretical modeling. Entrance disturbances are introduced by small beads, and localized turbulent patches can be triggered at low Reynolds numbers (*Re*). High turbulence intensity represents strong ability of perturbation spread, and a maximum turbulence intensity is found for every test case as *Re* ≥ 950, where the turbulence fraction increases abruptly with *Re*. Skewness can reflect the velocity defects of localized turbulent patches and is revealed to become negative when *Re* is as low as about 660. It is shown that the third-order moments of the midplane streamwise velocities have minima, while the corresponding forth-order moments have maxima during the transition. These kinematic extremes and different variation scenarios of the friction coefficient during the transition are explained with an intermittent structure model, where the robust localized turbulent structure is simplified as a turbulence unit, a structure whose statistical properties are only weak functions of the Reynolds number.

## 1. Introduction

Plane Poiseuille flow (PPF), the flow driven by a pressure gradient between two parallel plates, displays a parabolic velocity profile at its laminar state and becomes linearly unstable when the Reynolds number is larger than the critical value, $Re_{c}$ = 5772 [1]. The Reynolds number (*Re*) is defined as 1.5$U_{b}^{*}h^{*}/\nu^{*}$, where $U_{b}^{*}$ is the bulk velocity, $h^{*}$ is the half-channel height, and $\nu^{*}$ is the kinematic viscosity of the fluid. In practice, PPF may become turbulent at much lower Reynolds numbers than $Re_{c}$ due to the subcritical transition, where the finite-amplitude disturbances are necessary and the nonlinear effect cannot be ignored [2,3,4]. Davies and White [5] measured the friction coefficient of PPF with different aspect ratios of the cross-sections in a wide range of Reynolds numbers. It was shown that the critical Reynolds number of the subcritical transition increases with the ratio between the entrance length and the channel height, and it remains at 667.5 when the entrance length is larger than 108*h*. Patel and Head [6] found experimentally that PPF remained laminar as *Re* < 1035, and intermittent bursts occurred as 1035 < *Re* < 1350. Later experiments by Nishioka and Asai [7] confirmed that the turbulent state could hardly be sustained as *Re* < 1000. Based on flow visualizations, Carlson et al. [8] found that the orifice jet on the wall can trigger turbulent spots when *Re* is about 1000, and when *Re* < 840, the turbulent spots cannot be formed completely and decay eventually. Later experimental, theoretical and numerical works were mainly focused on the turbulent spots as *Re* > 1000 [9,10,11,12,13,14]. According to the experiments of Alavyoon, et al. [15], the complete spot cannot be triggered by orifice jet if *Re* < 1100. Recently, turbulent stripes or bands were revealed by numerical simulations for *Re* ≥ 1070 [16,17] and were observed by flow visualizations [18]. It was found experimentally that the turbulent bands would break as *Re* < 1275, and the flow remained stable and laminar at *Re* = 975 [19].

Based on numerical simulations within a tilted long and narrow domain, Tuckerman found turbulent band structures as *Re* > 850 [20]. By applying entrance disturbances and flow visualization techniques, Sano and Tamai [21] obtained the turbulence fraction at a range of Reynolds numbers and defined a threshold of 830 for the transition by fitting the data with the Directed Percolation (DP) model. According to their experimental data, however, the turbulence fractions are not zero as *Re* < 830. Recent numerical simulations revealed that the DP power law is retrieved only when *Re* is above 924, and relaminarization will occur in the long-time limit as *Re* < 700 [22]. Numerical simulations in large domains showed that localized turbulent bands can be obtained when *Re* is reduced to 720 [23]. Further numerical investigations illustrated that the isolated turbulent band, a single banded coherent structure surrounded by a large laminar region, can obliquely extend at moderate Reynolds numbers but will decay eventually as *Re* < 665 [24]. This threshold Reynolds number, in fact, agrees with the experimental observation by Davies and White [5]. It is tested that the periodic turbulent band can sustain as *Re* < 750, though band breaking and band reconnection may occur [25]. Recently, the turbulent bands were observed at *Re* = 750 by flow visualization [26], and the mean growth rate of turbulence fraction was found to become positive at *Re* ≈ 650 [27,28]. Therefore, in the literature, there have been discrepancies on the threshold Reynolds number for sustained turbulence in channel flows.

Besides the turbulence fraction, other statistical parameters are studied as well for the transitional channel flows. Turbulence intensities at the channel center are measured and are found to increase rapidly around *Re* = 1050, reach a peak at *Re* = 1140, and then gradually decrease with increasing *Re* [29]. The intermittent low- and high-drag events are investigated numerically and experimentally [30,31,32], and it is found that the conditionally averaged Reynolds shear stress is higher than the mean value during the low-drag events [33]. Based on simulations of channel flows with constant pressure gradients, a linear correlation for the wall shear stress is observed between its kurtosis and its skewness squared [34]. It is known that high-order moments of velocity derivatives are important to understand the non-Gaussian behavior of turbulence [35], and the intermittency is a key concept to develop turbulence model for the transitions of incompressible, supersonic, and hypersonic boundary layer flows [36]. However, the study on the relation between the turbulence fraction and the high order moments of velocities in the transitional channel flows is still rudimental.

In this paper, a wind channel with a large width-to-height ratio is used to study the subcritical transition of PPF, and its configuration is introduced in Section 2. In Section 3, it is revealed that the turbulence intensity and the kurtosis of midplane streamwise velocity reach their maxima while the skewness has a negative minimum during the transition. Furthermore, an intermittent structure model is constructed to describe the velocity features of localized turbulent structures and derive theoretically the high-order moments of midplane velocity and the friction coefficient, which are shown to be consistent with the experimental data. In Section 4, conclusions are presented.

## 2. Experimental Apparatus and Methods

### 2.1. Wind Channel

**Figure 1:**
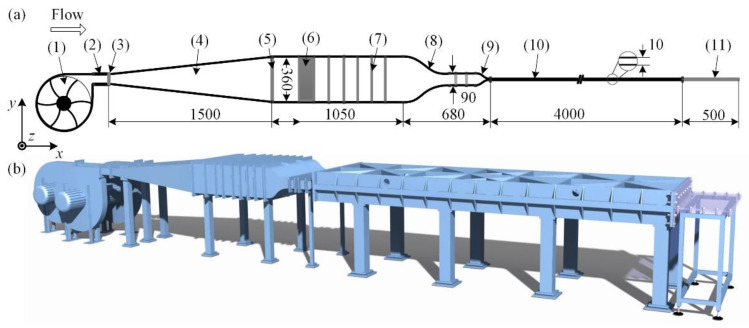
Sketch of the wind channel. (**a**) The components of the wind channel: (1) centrifugal fan, (2) soft connection, (3) fine damping screen, (4) expansion section, (5) perforated screen, (6) honeycomb, (7) screen, (8) first contraction, (9) second contraction, (10) first working section (steel), and (11) second working section (tempered glass). Unit of length, mm; (**b**) 3D drawing of the wind channel.

The open-circuit wind channel used in the experiment is shown in Figure 1. The length, width, and height 2h* of the working section are 4.5, 1.0, and 0.01 m, respectively. The flow is driven by three centrifugal fans with 1.5 kW induction motors, and the midplane velocity in the working section is controlled by a frequency converter to vary between 0.4 and 28 m·s^−1^. In order to isolate the vibration noise generated by the centrifugal fans, a soft connection is attached just in front of the expansion section. A perforated screen and 5 stainless-steel screens are mounted near the honeycomb layer to stabilize the flow and decrease the turbulence intensity. Two contractions with the contraction ratios of 4:1 and 9:1 are used to further reduce the turbulence intensity to a level less than 0.2%.

The channel walls of the first working section are polished to achieve a surface roughness less than 15 μm and are supported by steel frames, to avoid deflection. According to the finite element analyses, the maximum deflection of the whole test section is less than 3.7 μm. The second part is a transparent test section with a length of 0.5 m, granting optical access to the Particle Image Velocimetry (PIV) setup. Two 10-mm-thick side walls are sandwiched between the top and the bottom walls, and the error of channel height in the working section is less than 0.01 mm. In all experiments, the ambient temperature variation is less than 2 degrees centigrade. For non-dimensionalization, the half channel height h* and the time averaged velocity at the midplane $U_{c}^{*}$ are chosen as the characteristic length and velocity, respectively, and the dimensionless parameters have no superscript. For laminar flows, $U_{c}=1.5U_{b}$. The origin of the coordinates lies at the entrance center of the working section, and the dimensionless *x*, *y*, and *z* represent the streamwise, the wall-normal, and the spanwise directions, respectively.

### 2.2. Experimental Methods and Validations

Eighteen static pressure holes with 0.5 mm diameter are drilled on the lower wall along the line *z* = 0 with an interval of *l* = 200 mm, and the first hole is located at 300 mm from the entrance of the working section. Consequently, the pressure gradient along the streamwise direction can be monitored by using micro differential pressure transducers (Alpha M168, range: 0~25 Pa, accuracy: ±0.25% FS). A low-noise hot-wire anemometer (HWA, Dantec StreamLine Pro.) with 3 channels is used to measure the velocity with a relative error less than 1.5%. The stainless-steel probe stem is mounted on a two-dimensional traversing mechanism with a positioning resolution of 5 μm. In order to minimize the interference, the probes are inserted through the outlet of the working section.

**Figure 2:**
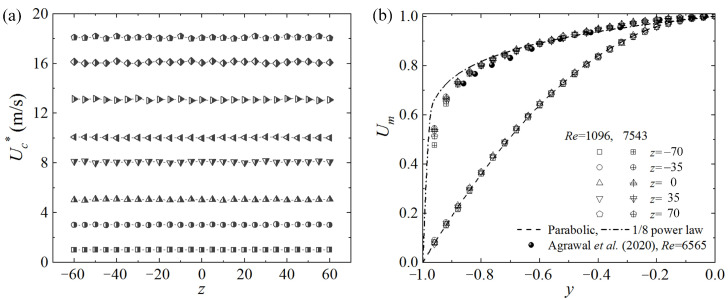
Streamwise velocities measured at *x* = 780. (**a**) Spanwise distributions of the time averaged velocity in the midplane $U_{c}^{*}$, and (**b**) the time averaged velocity profiles at different spanwise positions. The measurements of Reference [33] are added in (**b**) as references.

We checked that, except the region very close to the entrance, the streamwise pressure gradients remained constant at low Reynolds numbers and agreed with the theoretical values for laminar PPF as reflected by the friction coefficients, which are discussed in Section 3.1. As shown in Figure 2a, the uniform distribution of, $U_{c}^{*}$, in the spanwise direction indicates that the velocity field in the central part of the cross-section is hardly affected by the sidewalls. When the flow is laminar at *Re* = 1096, it is shown in Figure 2b that the velocity profiles at five different spanwise positions agree well with the theoretical parabolic distribution. When *Re* is increased to 7543, the time averaged velocity profiles are all close to the 1/8 power law curve, confirming that the sidewall effect is still negligible in the central region. Without the entrance artificial disturbances, it is checked that the flow can remain laminar for *Re* up to 3500, and hence the present setup is appropriate to study the subcritical transition of PPF.

Nine plastic beads evenly spaced with an interval of 100 mm along a thin iron wire are placed at the centerline of channel inlet to introduce entrance disturbances. Different bead diameters, D*, and wire diameters, d*, are used in four cases and are listed in Table 1.

## 3. Results and Discussions

### 3.1. Friction Coefficient

**Figure 3:**
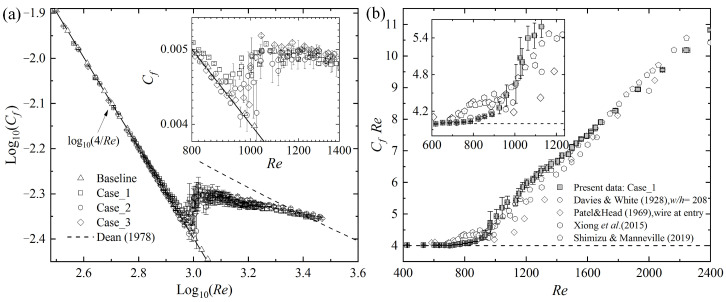
(**a**) The friction coefficient, *C_f_*, as a function of *Re*. The previous experimental and numerical data are illustrated in (**b**) for references.

The friction coefficient $C_{f}=8\left(h^{*}\frac{dP^{*}}{dx^{*}}\right)/\left(9\rho^{*}U_{b}^{*2}\right)$ is measured at different Reynolds numbers, with different entrance disturbances, where $dP^{*}/dx^{*}$ is the mean pressure gradient calculated based on the pressure difference between *x* = 660 and 740, and the bulk velocity, $U_{b}^{*}$, is obtained from the mean velocity profile. $C_{f}$ is calculated for every 10-s sample, and the averaged $C_{f}$ for 20 samples (totally 10^4^~10^5^ time units at the transition stage) are shown in Figure 3, where the error bars represent the standard deviation. It is shown that when *Re* < 600 or there are no entrance artificial disturbances (Baseline), the present experimental data agree well with the laminar value $C_{f}=4/Re$. The previous results [5,6,22,24] are shown as well for references. When *Re* is greater than 1750, $C_{f}$ data for different entrance disturbance cases tend to agree with the “optimum log-law” labeled by the dashed line for developed turbulence, where $Re=\sqrt{\frac{2}{C_{f}}}\exp\left[0.41\left(\sqrt{\frac{8}{9C_{f}}}-2.4\right)\right]$ [22,37]. During 950 < *Re* < 1010, $C_{f}$ in three disturbed cases increases abruptly, reflecting a strong development of turbulence. As shown in the inset of Figure 3b, such an abrupt increase of $C_{f}$ occurs as well in the previous direct numerical simulations, where the turbulent band split occurs, i.e., parallel split to form a new band parallel to the original one and transverse split to sprout new branch (as shown by Figure 6 of Reference [24]). Recent systematical simulations [22] revealed that the transition from “one-sided” (all localized turbulent bands point to the same direction) to “two-sided” (the bands may grow in different directions) propagations takes place at *Re* ≈ 924. By simulations in tilted slender domains, a critical Reynolds number is defined as 950, where the statistically estimated mean lifetimes for band decay and splitting coincide with each other [38]. All of these numerical results explain, to some degree, why $C_{f}$ increases abruptly as *Re >* 950.

### 3.2. Turbulence Intensity and Pressure Turbulence Intensity

**Figure 4:**
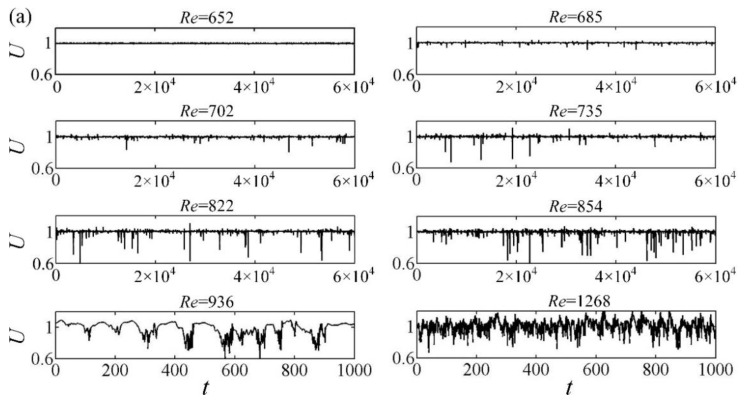

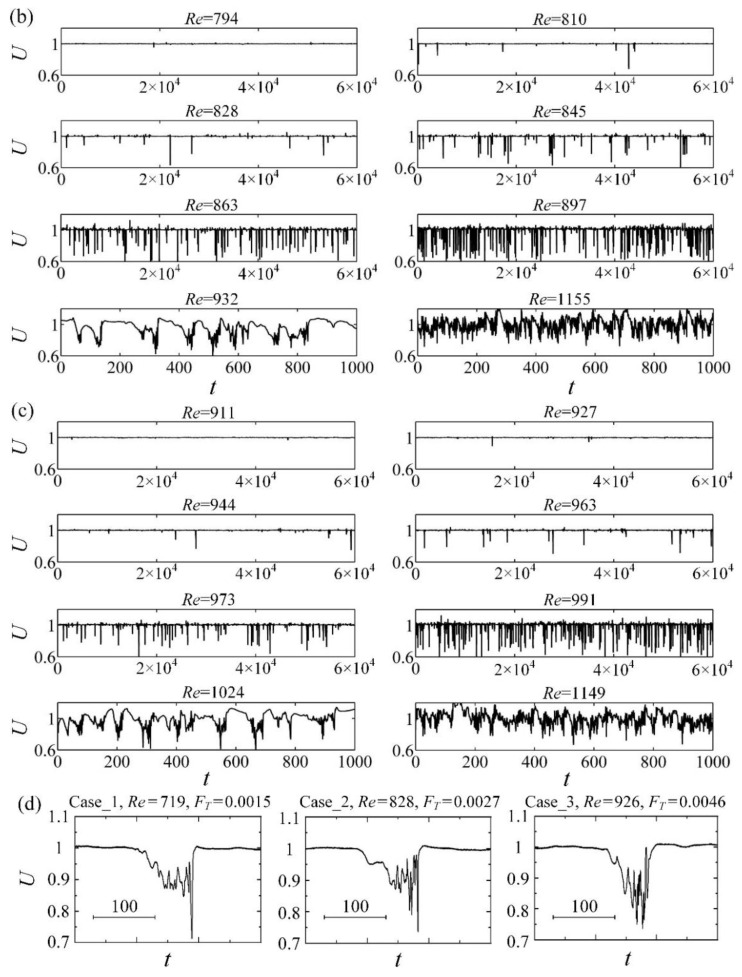
The time series of velocity, *U*, measured at (*x*, *z*) = (780, 0) for (**a**) Case_1, (**b**) Case_2, and (**c**) Case_3. Typical signals of localized turbulent structures for different cases at different *Re* and turbulence fraction, *F_T_*, are shown in (**d**).

The time series of the streamwise velocity, U, obtained at the midplane by HWA are just straight lines superimposed by background noise at low Reynolds numbers, e.g., *Re* = 652 in Figure 4a. When a turbulent band or spot passes through the measuring point, the time series show a velocity defect, i.e., the midplane streamwise velocity decreases first along with the time, then oscillates strongly with high frequencies before increasing abruptly to recover its laminar level. The velocity fields of the spots and turbulent bands are measured by PIV, and their consistencies with the direct numerical simulations are confirmed and shown in [39]. The present study mainly focuses on the statistical kinematic and dynamic properties of the transitional flow. It is shown in Figure 4d that the widths and amplitudes of the velocity defects are comparable for different entrance disturbances and different Reynolds numbers, indicating that the statistical properties of localized structures are weak functions of *Re* and external disturbances during the transition. Such a streamwise velocity defect appears more and more frequently with the increase of *Re*, as shown in Figure 4.

**Figure 5:**
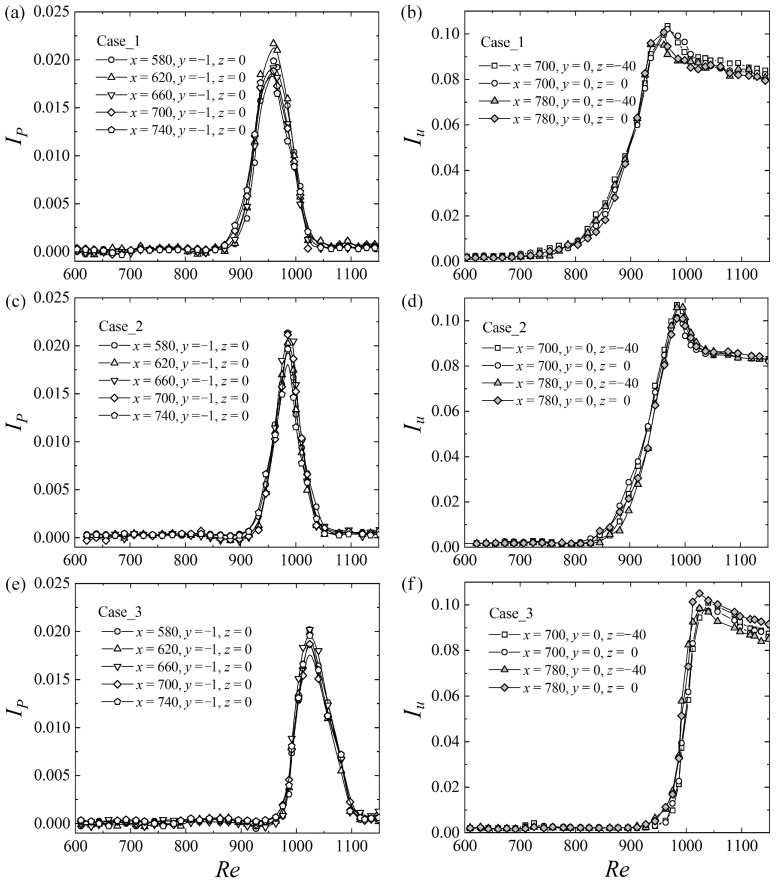
Pressure turbulence intensity, *I_P_* (left column), and turbulence intensity, *I_u_* (right column) measured at different locations. (**a**,**b**), (**c**,**d**), and (**e**,**f**) are for Case_1, Case_2, and Case_3, respectively.

The development of turbulence may be described by the turbulence intensity of streamwise velocity $I_{u}={\langle u^{2}\rangle }^{1/2}={\langle {\left(U-U_{c}\right)}^{2}\rangle }^{1/2}$ at the midplane (*y* = 0) and the pressure turbulence intensity $I_{P}=P_{rms}/\left(dP/dx\right)-{\left[P_{rms}/\left(dP/dx\right)\right]}_{r}$, where 〈 〉 means the time averaged quantity, and the subscripts *r* and *rms* represent a reference value and the root mean square. In this paper, ${\left[P_{rms}/\left(dP/dx\right)\right]}_{r}$ is the value at *Re* = 600, corresponding to a laminar flow with background noise. When *Re* is smaller than 850, $I_{P}$ remains a small value and is almost independent of the entrance disturbances, the downstream position, and the Reynolds number as shown in Figure 5a. When *Re* is larger than 850, $I_{P}$ of Case_1 increases obviously and reaches a peak at about *Re* = 950 before decreasing. The corresponding *Re* of $I_{P}$ peaks for Case_2 and Case_3 is around 980 and 1020, respectively. In the right column of Figure 5, it is shown that the turbulence intensity, $I_{u}$, has peak values at the same *Re* as $I_{P}$ for all three cases. The existence of these peaks is explained in Section 3.5, with an intermittent structure model.

### 3.3. Skewness and Kurtosis

**Figure 6:**
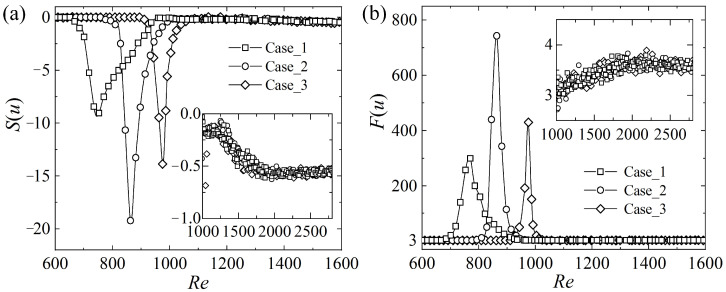
(**a**) Skewness and (**b**) kurtosis of the streamwise velocity measured at (*x*, *y*, *z*) = (780, 0, 0) for different disturbance cases and Reynolds numbers.

Though $I_{P}$ and $I_{u}$ reflect the mean levels of fluctuation amplitudes or strengths, they cannot describe the intermittency and asymmetry of the signals. In this subsection, the skewness $S\left(u\right)=\;\langle u^{3}\rangle /{\langle u^{2}\rangle }^{3/2}$ is calculated based on the streamwise fluctuation velocity, *u*, measured at the midplane, representing the asymmetric distribution of the velocity. The kurtosis or flatness $F\left(u\right)=\langle u^{4}\rangle /{\langle u^{2}\rangle }^{2}$ is computed as well, reflecting the intermittency and the deviation from the random distribution. At low Reynolds numbers, the laminar velocity signal mixed with the background white noise conforms to the normal distribution, and hence *S*(*u*) = 0 and *F*(*u*) = 3. When the localized turbulent spots or bands emerge intermittently in the flow, the velocity defects appear, leading to a negative skewness and a positive flatness, e.g., *Re* < 700 for Case_1 shown in Figure 6, while the corresponding turbulence intensity (Figure 5) and the friction coefficient (Figure 3) remain nearly unchanged. Specially, it is shown in Figure 6 that the skewness and the kurtosis reach a minimum and a maximum during the transition, respectively, and the corresponding underlying mechanisms are discussed in Section 3.5.

The transition process is triggered by the entrance disturbances, the abundant vortex structures shed from the beads placed at the inlet. It has been shown that, at $Re_{D}\;$= 3700 (based on the free-stream velocity and the sphere diameter *D*), the turbulence intensity, $I_{u}$, along the wake centerline of a sphere quickly reduces to 0.05 at *x*/*D* = 12 [40]. Based on the centerline velocities measured for *Re* = 600~1200, the corresponding $Re_{D}$ for the present inlet beads can be estimated to be 720~1920. Considering that the working section is 500*D*~666*D* long, the strong turbulence intensity, $I_{u}$, around 0.1, as shown in Figure 5, should be caused by the localized turbulent patches triggered by the remnants of the bead wakes rather than the remnants themselves. According to Figure 6, the Reynolds number intervals where the skewness and the kurtosis deviate from the normal distribution are [660,960], [780,1000], and [910,1060] for Case_1, Case_2, and Case_3, respectively. It is interesting to note that the upper limits of these *Re* intervals are close to the corresponding peak *Res* for $I_{P}$ and $I_{u}$ shown in Figure 5. The lower limits indicate the onset of turbulence, and the minimum lower limit of tested cases is about 660, which is consistent with the threshold determined numerically for the oblique turbulent bands [24,25] and the value obtained by flow visualization [27]. In numerical simulations, the computation may last long enough, e.g., ~10^4^ time units, to observe the transient growth and eventual decay of the patterns near the critical state, while, in experiments, the channel length is limited and the traveling turbulent patches may grow transiently but have no time to experience the final decay. This factor may cause a mild underestimate of the threshold value in experiments. It is shown in the insets of Figure 6 that, when *Re* > 1100 and $F_{T}$ is close to 1, the skewness and the kurtosis of streamwise velocity continue to evolve, deviating from 0 and 3 (the values for white Gaussian noise) and remain at about −0.5 and 3.5 after *Re* > 1750, respectively, the values for fully developed turbulence [41]. Consequently, the threshold for fully developed turbulence may be defined as *Re* ≈ 1750.

### 3.4. Turbulence Fraction

**Figure 7:**
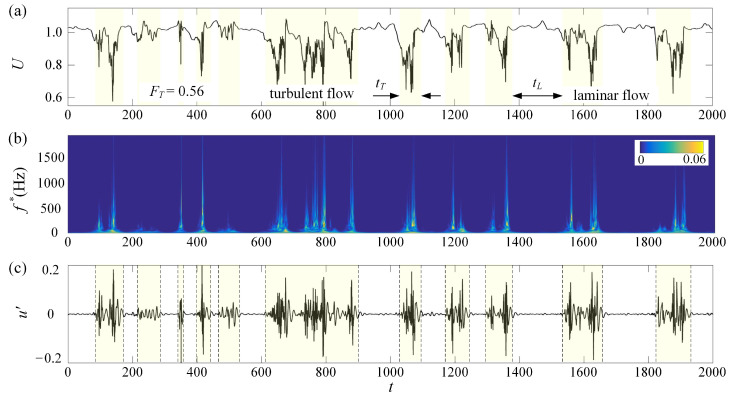
(**a**) The time series of streamwise velocity *U* measured at (*Re*, *x*, *y*, *z*) = (935, 780, 0, 0) for Case_1 and (**b**) its wavelet power spectrum. (**c**) The high-frequency component, *u*′, after high-pass filtering of the signal shown in (**a**). Localized turbulent patches are marked with shadowed areas in (**a**,**c**).

**Figure 8:**
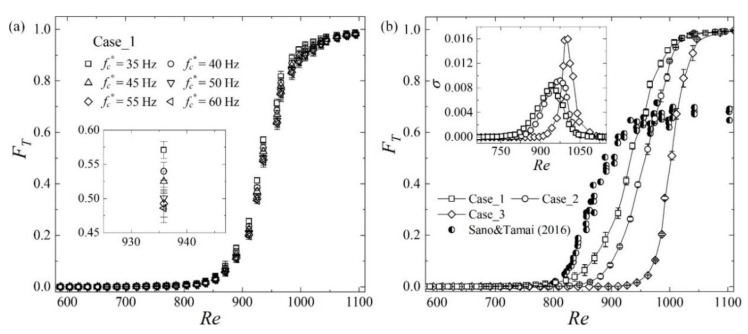
(**a**) $F_{T}$ calculated with different cutoff frequencies, $f_{c}^{*}$, for Case_1, and (**b**) data calculated with $f_{c}^{*}=45\;\mathrm{Hz}$ for different entrance disturbances. Inset of (**b**): the growth steepness *σ* versus *Re*.

An important parameter to describe the pattern evolution and intermittency during the subcritical transition is the turbulence fraction, $F_{T}$, whose determination relies on the identification of the boundaries between the laminar and the turbulent regions. Different from the previous experiments, where $F_{T}$ was mostly calculated based on flow visualization images, in this paper, the time series of velocity are used to define $F_{T}$ as $F_{T}=\sum^{\;}t_{T}/t_{Total}$, where $t_{T}$ and $t_{Total}$ are the turbulent period and the total sampling time, respectively. As shown in Figure 7a, the time series of the midplane streamwise velocity includes many velocity defects, which correspond to the traveling localized turbulent patches and include high-frequency components, as illustrated by the wavelet power spectrum shown in Figure 7b. Consequently, high-pass filtering is used to extract these components, as shown in Figure 7c, whose time intervals are defined as the turbulent period, $t_{T}$. Different cutoff frequencies, $f_{c}$, are tested, and the corresponding $F_{T}$ values vary in the same trend, as shown in Figure 8a, though a higher $f_{c}$ leads to a lower $F_{T}$. By comparing Figure 7a,c, the cutoff frequency of 45 Hz is found to capture the turbulent periods reasonably well, and hence is used in the following analyses.

$F_{T}$ shown in Figure 8 is computed from the midplane streamwise velocity signals sampled at six locations, i.e., (*x*, *z*) = (700, −40), (700, −20), (700, 0), (780, −40), (780, −20), and (780, 0). Each time series lasts 2000 s (10^5^~10^6^ time units at the transition stage), and the error bar represents the standard deviation. As *Re* < 850, the localized patches are far from each other, as shown in Figure 4, and $F_{T}$ increases slowly with *Re* and is less than 0.1 for all three cases. When *Re* is larger than 1050, the localized turbulent structures almost occupy the whole flow field and are arranged nearly side by side, as shown by the case of *Re* = 1155 in Figure 4b, and hence $F_{T}$ is close to 1, as shown in Figure 8. The growth steepness $\sigma =dF_{T}/dRe$ is calculated and is found to reach its maxima (as shown in the inset of Figure 8b) at *Re* = 950, 975, and 1005 for Case_1, Case_2, and Case_3, respectively, where $F_{T}$ is around 0.6. It is interesting to note that the Reynolds numbers of the *σ* peaks are almost the same as those of the $I_{P}$ and $I_{u}$ peaks shown in Figure 5, confirming the intrinsic relation between the turbulence intensity and the growth steepness of the turbulence fraction.

According to Table 1, the beads’ diameters are different for Case_1 and Case_2, representing different localized disturbance intensities, and the wire diameter of Case_3 is about one order larger than that of Case 1, denoting different entrance disturbance forms, i.e., the entrance disturbances of Case_3 are more uniform in the spanwise direction due to the vortex shedding of the thicker wire. As shown in Figure 8b, $F_{T}$ data for different entrance disturbances vary in the same manner but do not collapse with each other as 850 < *Re* < 1050, reflecting the sensitivity of transition to the external forcing, and the reason lies in several aspects. Firstly, $F_{T}$ data collapse will occur when $F_{T}$ is a single valued function of *Re*, e.g., at laminar state or the equilibrium state, which is found to be retrieved only as *Re* > 924 in long-term simulations [22]. In other words, when the upstream or initial disturbances are different, $F_{T}$ may be different from case to case as *Re* < 924 even for simulations with the same computational configurations, e.g., domain size and mode numbers. Secondly, in reality, the lengths of experimental channels are finite, and at moderate Reynolds numbers, the turbulent structures may have no enough time to spread completely before leaving the outlet. Consequently, $F_{T}$ will depend on the entrance disturbances. Thirdly, the effectiveness to trigger the transition are different for different types of perturbations. The turbulence fractions obtained based on flow visualization by Sano and Tamai [21] are shown in Figure 8b, as well, and are different from the present data: $F_{T}$ does not increase with *Re* as *Re* > 1000 but maintain at about 0.7. In Sano and Tamai’s experiments, turbulent flow was excited in a buffer box by a grid and injected from the inlet, and hence the entrance perturbations occupied the span of the channel and are different from the localized disturbances used in this paper. In addition, different approaches applied to identify the laminar–turbulent boundaries and different data (e.g., the two-dimensional images of flow visualization and the one-dimensional velocity series measured by HWA) may lead to different $F_{T}$ values, as well. 

### 3.5. Intermittent Structure Model

**Figure 9:**
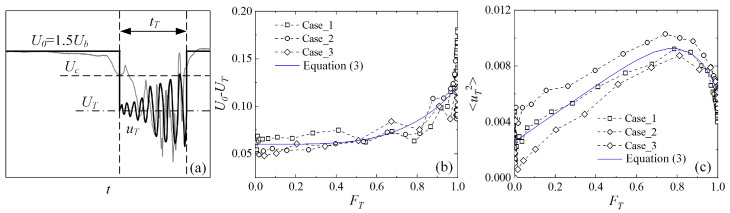
(**a**) The simplified velocity signal (thick solid line) of the intermittent structure model at midplane, and the time averaged (**b**) $U_{0}-U_{T}$ and (**c**) $\langle u_{T}^{2}\rangle$ sampled at the midplane during the turbulent periods. A measured midplane velocity signal is shown in (**a**) by the gray curve for a reference.

In order to understand the peaks and valleys of turbulence intensity and high-order moments during the transition, an intermittent structure model is constructed as follows. For convenience, the characteristic velocity is chosen as $1.5U_{b}^{*}$ instead of $U_{c}^{*}$ in this subsection. The velocity during the turbulent period is decomposed into two parts: the turbulent mean velocity, $U_{T}$, representing the behavior of low-frequency and large-scale structures, and the turbulent perturbation velocity, $u_{T}$ (relative to $U_{T}$), denoting the high-frequency and small-scale components. $U=U_{T}+u_{T}$, and it is assumed that $u_{T}$ satisfies Gaussian distribution, i.e., the time averaged values $\langle u_{T}\rangle$ = 0, $\langle u_{T}^{3}\rangle$ = 0, and $\langle u_{T}^{4}\rangle$ = 3${\langle u_{T}^{2}\rangle }^{2}$, but its temporal and spatial distribution is strongly asymmetric and aperiodic just like the measured velocity (gray curve) shown in Figure 9a. Assuming that $U_{T}$ and $\langle u_{T}^{2}\rangle$ are the same for all localized turbulent patches in a given case and $F_{T}$ is known, it can be derived that the mean velocity $U_{c}=U_{0}-F_{T}\left(U_{0}-U_{T}\right)$ and the fluctuation velocity relative to $U_{c}$ is as follows:

(1) $$u=U-U_{c}=\{\begin{array}{l} F_{T}\left(U_{0}-U_{T}\right),\;\;\;\;\;\;\;\mathrm{laminar}\;\mathrm{periods},\\ \left(U_{0}-U_{T}\right)\left(F_{T}-1\right)+u_{T},\;\;\;\mathrm{turbulent}\;\mathrm{periods}. \end{array}$$

Consequently, the turbulence intensity and the high-order moments can be derived as follows:

(2) $$\{\begin{array}{c} I_{u}=\frac{\sqrt{\langle u^{2}\rangle }}{U_{c}}=\frac{\sqrt{F_{T}\left(1-F_{T}\right){\left(U_{0}-U_{T}\right)}^{2}+\langle u_{T}{}^{2}\rangle F_{T}}}{U_{0}-F_{T}\left(U_{0}-U_{T}\right)}\\ \langle u^{3}\rangle =3F_{T}\left(U_{0}-U_{T}\right)\langle u_{T}{}^{2}\rangle \left(F_{T}-1\right)-F_{T}{\left(U_{0}-U_{T}\right)}^{3}\left(2F_{T}{}^{2}-3F_{T}+1\right)\\ \langle u^{4}\rangle =F_{T}\left(1-F_{T}\right)\left(1-3F_{T}+3F_{T}{}^{2}\right){\left(U_{0}-U_{T}\right)}^{4}+3F_{T}{\langle {u_{T}}^{2}\rangle }^{2}+6F_{T}\langle {u_{T}}^{2}\rangle {\left(F_{T}-1\right)}^{2}{\left(U_{0}-U_{T}\right)}^{2} \end{array}$$

$U_{T}$ is estimated by the mean value of low-pass filtered midplane velocity during the turbulent periods at each *Re*, and the cutoff frequency, $f_{c}$, used for the filtering is the same as those used for calculating $F_{T}$. It is shown in Figure 9 that $U_{0}-U_{T}$ increases with $F_{T}$, while the variance $\langle u_{T}^{2}\rangle$ increases first then decreases with the growth of $F_{T}$, reflecting the fact that the localized turbulent structures are influenced to some degree by the entrance disturbances, $F_{T}$, and then *Re*. $U_{0}-U_{T}$ and $\langle u_{T}^{2}\rangle$ may be fitted as follows:

(3) $$U_{0}-U_{T}=0.06\left(1+F_{T}^{4}\right),\;\;\langle u_{T}^{2}\rangle =0.0026+0.01\left(F_{T}-0.64F_{T}^{7}\right),$$

which are shown in Figure 9b,c as solid curves.

According to the previous studies [42], the characteristics of localized turbulent bands, e.g., the band’s tilt angle, width, and convection velocity, do not change much during the transition. Similar properties are shown in Figure 4d, as well: The midplane velocity defects of localized turbulent structures are similar and not very sensitive to the Reynolds number, the entrance disturbances, and the turbulence fractions. Therefore, these localized turbulent structures may be simplified to a unified structure, whose statistical dimensionless properties are independent of time, $F_{T}$, and the initial or upstream disturbances. This unified structure is referred as turbulence unit hereafter. Consequently, $U_{0}-U_{T}$ and $u_{T}^{2}$ are chosen for mature structures and are set as the values when $F_{T}$ reaches 1, and then Equation (3) is simplified as follows:

(4) $$U_{0}-U_{T}=0.12,\;\;\;\langle u_{T}^{2}\rangle =0.006.$$

**Figure 10:**
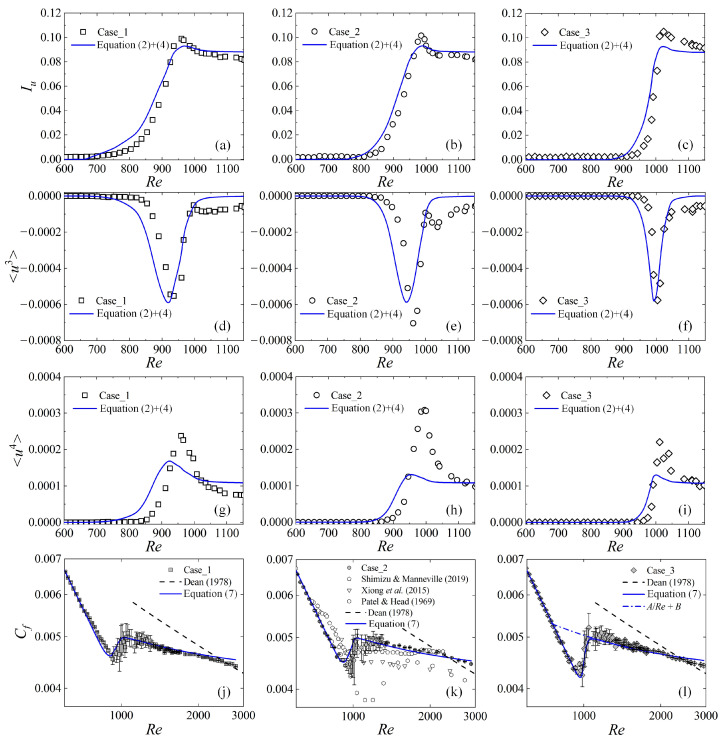
Turbulence intensity (**a**–**c**), the third (**d**–**f**) and the fourth (**g**–**i**) order moments of the midplane velocity, and the friction coefficient (**j**–**l**) for different disturbance cases. The symbols of different cases shown in (**a**–**i**) are experimental data measured at (*x*, *y*, *z*) = (780, 0, 0), and *C_f_* symbols shown in (**j**–**l**) are the same as those shown in Figure 3a. The solid curves are the results of the intermittent structure model.

For all three test cases, it is shown in Figure 10a–i by the solid lines that the main features of the second-, third-, and forth-order moments predicted by the model are consistent acceptably with the experimental results when the relations between $F_{T}$ and Re shown in Figure 8b are applied. The variance of the midplane streamwise velocity $\langle u^{2}\rangle$ is $F_{T}\left(1-F_{T}\right){\left(U_{0}-U_{T}\right)}^{2}+\langle {u_{T}}^{2}\rangle F_{T}$, where the contribution of fluctuations (the second term) increases with $F_{T}$, while the first term increases first and then decreases with $F_{T}$ due to the fact that the mean velocity, $U_{c}$, leaves $U_{0}$ for $U_{T}$, leading to a peak value of $\langle u^{2}\rangle$. Consequently, there exist peak values of $I_{u}$ and $\langle u^{4}\rangle$ during the transition. Furthermore, when $F_{T}$ is close to 1 and the flow field is nearly fully occupied by the localized turbulent structures, $U_{c}$ is almost as low as $U_{T}$, and $\langle u^{2}\rangle$ and $\langle u^{3}\rangle$ are close to $\langle {u_{T}}^{2}\rangle$ and $\langle {u_{T}}^{3}\rangle$, respectively. Therefore, at the late transition stage, $\langle u^{3}\rangle$ should be close to zero again, and then there must exist a minimum $\langle u^{3}\rangle$ during the transition. Similarly, the asymptotic values for $I_{u}$ and $\langle u^{4}\rangle$ should be finite ($\sqrt{\langle {u_{T}}^{2}\rangle }/U_{T}$ and $3{\langle {u_{T}}^{2}\rangle }^{2}$ in the model), just as shown by the experimental data in Figure 10. The consistencies of the model curves with the experimental data indicate that, not only the turbulence fraction, but also the characteristics of localized structures is required in order to describe properly the statistical properties of transitional flows.

Recently, it is found that, for a channel flow with constant pressure gradient, the kurtosis of the bulk velocity, which fluctuates during the transition and is represented by *Re*_b_ in the simulations [34], increases abruptly as the Reynolds number decreases to the threshold value. However, the kurtosis obtained in experiments is close to zero near the onset of turbulence, as shown in Figure 6. This discrepancy may be explained to some degree with the present model. Considering that, in simulations, the velocities in the laminar periods are as clean as the present model and have no background random noise, an inevitable factor in experiments, then when $F_{T}$ is close to 0, $\langle u^{4}\rangle \sim F_{T}$ while ${\langle u^{2}\rangle }^{2}\sim {F_{T}}^{2}$ according to Equation (2), and hence the kurtosis will increase sharply.

Next, we use this model to study the dynamic property. Considering a turbulence unit with volume, *V*, mean velocity, $U_{T}\left(y\right)$, and mean pressure, $P_{T}$, the perturbation velocities are $u_{T}$, $v_{T}$, and $w_{T}$, and then the volume averaged friction coefficient is obtained from the mean x-momentum equation:

(5) $$C_{fT}=-\frac{2}{V}\int^{\;}\frac{\partial P_{T}}{\partial x}\mathrm{dV}=-\frac{2}{ReV}\int^{\;}\frac{d^{2}U_{T}}{dy^{2}}\mathrm{dV}+\frac{2}{V}\int^{\;}\left[\frac{\partial \langle u_{T}^{2}\rangle }{\partial x}+\frac{\partial \langle u_{T}w_{T}\rangle }{\partial z}\right]\mathrm{dV}.$$

Note that $\int_{-1}^{1}\frac{\partial \langle u_{T}v_{T}\rangle }{\partial y}dy=0$. Since the velocity fluctuations are strongly asymmetric and there is nearly a velocity discontinuity at the later edge of time series (upstream edge) of the structure and the present model (Figure 9a), the Reynolds stresses, e.g., $\langle u_{T}^{2}\rangle$, are different at the upstream and the downstream edges of the turbulence unit. In fact, the Reynolds stresses of a localized turbulent band are aperiodic in both the streamwise and the spanwise directions, as shown by the disturbance velocity structures in Figure 2b of Reference [23], due to its oblique manner. Since the transition occurs at relatively high Reynolds numbers and the properties of turbulence unit are assumed to be weak functions of *Re*, $-\frac{2}{ReV}\int^{\;}\frac{d^{2}U_{T}}{dy^{2}}\mathrm{dV}$ may be expanded with 1/*Re* as $\frac{4}{Re}-\frac{2}{Re}\left(A_{0}+A_{1}\frac{1}{Re}+A_{2}\frac{1}{Re^{2}}+\ldots \right)$, where $\frac{4}{Re}$ corresponds to the laminar state, and the constants $A_{i}$ represent the contribution of mean flow modification. Similarly, the Reynolds stress term (the second term on the right hand side of Equation (5)) is expanded as $B_{0}+B_{1}\frac{1}{Re}+B_{2}\frac{1}{Re^{2}}+\ldots$, where the constants $B_{i}$ reflect the aperiodicity of the Reynolds stress. Consequently, Equation (5) can be expressed as follows:

(6) $$C_{fT}=B_{0}+\frac{1}{Re}\left(4-2A_{0}+B_{1}\right)+\frac{1}{Re^{2}}\left(B_{2}-2A_{1}\right)+\ldots =B+\frac{A}{Re}+O\left(\frac{1}{Re^{2}}\right),$$

where *A* and *B* are constants for the turbulence unit. For a transitional flow with a turbulence fraction, $F_{T}$, the total friction coefficient can be obtained as follows, after ignoring the higher orders terms in Equation (6):

(7) $$C_{f}=\left(1-F_{T}\right)\frac{4}{Re}+C_{fT}F_{T}=\left(1-F_{T}+\frac{A}{4}F_{T}\right)\frac{4}{Re}+F_{T}B.$$

It is shown in Figure 10j–l and that Equation (7) describes well the variations of $C_{f}$ data for different entrance disturbance cases when the measured relation between $F_{T}$ and *Re* are applied. *A* and B are determined by fitting the data between *Re* = 1300 and 2000 as 0.78 and 0.00426, respectively.

At the initial and middle stages of transition, $C_{f}$ may have different variation scenarios. If the external disturbances are not effective to trigger the turbulent patches and the transition starts at high Reynolds numbers, $\left(1-F_{T}+\frac{A}{4}F_{T}\right)\frac{4}{Re}$ may become smaller than $F_{T}B$ after a short Re range, and then there will be a stage where $C_{f}$ increases with $F_{T}$ and *Re*, as shown in Figure 10. Note that *A* < 4 and $\left(1-F_{T}+\frac{A}{4}F_{T}\right)\frac{4}{Re}$ decreases with the increase of $F_{T}$ and *Re*. Consequently, there will be a maximum of $C_{f}$ during the transition as illustrated by the present data shown in Figure 10l and the data of Patel and Head [6] shown in Figure 10k. If the transition begins at low Reynolds numbers, the variation of $\left(1-F_{T}+\frac{A}{4}F_{T}\right)\frac{4}{Re}$ may be comparable with that of $F_{T}B$. Depending on the variation feature of $F_{T}$, the stage of $C_{f}$ growth may be short or even disappear, and a $C_{f}$ plateau may appear, where $C_{f}$ remains nearly constant in a finite range of *Re*. The $C_{f}$ plateaus were observed in the previous numerical simulations [22,24,34] and are shown in Figure 10k for references. According to Equation (7), provided that the decrease of $\left(1-F_{T}+\frac{A}{4}F_{T}\right)\frac{4}{Re}$ is balanced by the rise of $F_{T}B$, $C_{f}$ will keep constant, though this constant value may be different for different entrance or initial disturbances, domain sizes, and computational periods. At the late stage of transition, $F_{T}$ tends to 1, and $C_{f}$ is close to A/Re+B according to Equation (7) and then decreases with *Re*. The dashed lines in Figure 10j–l, $Re=\sqrt{\frac{2}{C_{f}}}\exp\left[0.41\left(\sqrt{\frac{8}{9C_{f}}}-2.4\right)\right]$, represent the fully developed turbulence [22,37], where the Reynolds stresses are assumed to be uniform in the streamwise direction. According to the experiments, $F_{T}$ is close to 1 as *Re* > 1100, but $C_{f}$ still deviates from the dashed line as *Re* < 1750, indicating a moderately developed turbulent state. By extrapolating A/Re+B to the laminar value 4/Re, as shown by the dot-dash line in Figure 10l, we get *Re* = 756, corresponding to an asymptotic threshold for the moderately developed turbulence.

## 4. Conclusions

In this paper, the subcritical transition of channel flow is studied experimentally and theoretically. A pressure turbulence intensity is defined to describe the pressure fluctuations, and it is found that both the pressure and the velocity turbulence intensities reach maxima at the same Reynolds number during the transition, where the turbulence fraction is about 0.6 and both the friction coefficient and the turbulence fraction increase abruptly with *Re*. The velocity defect of localized turbulent structure leads to a negative skewness, and for all tested cases, the smallest *Re* where the skewness of the midplane velocity starts to be negative is about 660. Since the onset of turbulence depends on not only the intensities but also the forms of initial or upstream disturbances, the high-order moments of fluctuations are better markers for the start of transition than the turbulence intensity or fluctuation kinetic energy, and hence should be considered in the future transition control strategies.

According to the experimental data, there exist maxima of the turbulence intensity and the forth-order moment of the midplane streamwise velocity and a negative minimum for the third-order moment. At the late stage of transition, the third-order moment decreases to a low level, and the turbulence intensity and the forth-order moment remain finite values. These phenomena are explained with an intermittent structure model, where the robust localized turbulent structure is simplified as a turbulence unit. In addition, different variation behaviors of the friction coefficient are explained by this model, as well, mainly in terms of the turbulence fraction and the aperiodic distribution of Reynolds stress in the localized turbulent structures, and the latter factor should be considered in the future transition modelling.

## Figures and Tables

**Table 1:** Dimensions of the entrance disturbances.

	Baseline	Case_1	Case_2	Case_3
D* (mm)	/	8	6	8
D*/h*	/	1.6	1.2	1.6
d* (mm)	/	0.2	0.2	1.5
d*/h*	/	0.04	0.04	0.3
